# Molecular Characterization of Aminoglycoside-modifying Enzymes (AMEs)in Aminoglycoside-Resistant *Staphylococcus aureus*: A Cross-sectional Study in Northeastern Iran

**DOI:** 10.30699/ijp.2024.2038509.3342

**Published:** 2025-01-10

**Authors:** Malihe Naderi, Neda Yousefi Nojookambari, Somayeh Talebi, Mohammad Reza Mohammadi, Sajjad Yazdansetad

**Affiliations:** 1 *Infectious Diseases Research Center, Golestan University of Medical Sciences, Gorgan, Iran*; 2 *Hiroshima Institute of Life Sciences, 7-21, Nishi Asahi-Machi, Minami-ku, Hiroshima-shi, Hiroshima, 734-0002 Japan*; 3 *Department of Microbiology, School of Medicine, Shahid Beheshti University of Medical Sciences, Tehran, Iran*; 4 *Department of Bacteriology, Faculty of Medical Sciences, Tarbiat Modares University, Tehran, Iran*; 5 *Department of Biology, Faculty of Basic Sciences, Imam Hossein Comprehensive University, Tehran, Iran*

**Keywords:** AMEs-encoding genes, Aminoglycoside, Drug resistance, FemA; Staphylococcus aureus

## Abstract

**Background & Objective:**

The resistance genes encoding aminoglycoside-modifying enzymes (AMEs) are now widely prevalent in different populations of* Staphylococcus aureus*. The study aimed to determine the frequency of AMEs-encoding genes in clinical isolates of *S. aureus*.

**Methods:**

A total of 105 *S. aureus* isolates were obtained from the different clinical samples; and then were identified by conventional biochemical tests. The antibiotic resistance patterns of the isolates were characterized by the agar disk diffusion method. The distribution of the AMEs and *femA* genes was determined by conventional and multiplex PCR.

**Results:**

The aminoglycoside resistance rates of kanamycin, tobramycin, gentamicin, amikacin, and netilmicin were 47.6%, 46.6%, 45.7%, 45.7%, and 26.6%, respectively. 16.1% and 1.9% of isolates were MDR and XDR phenotypes, respectively. 21.9% of *S. aureus* isolates harbored the *femA* gene and were determined as methicillin-resistant *S. aureus* (MRSA) clones. The *aac(6')/aph(2'')* was the most prevalent (47.8%) AME-encoding gene in aminoglycoside-resistant *S.* *aureus*, followed by *ant(4')-Ia *(30.4%) and *aph(3')-IIIa *(21.7%).

**Conclusion:**

Our study demonstrated that the coexistence of several AMEs and the spread of the resistance determinants like *femA* in *S. aureus* clinical isolates are alarming and may contribute to the broadening of aminoglycoside resistance spectra and limit treatment options for staphylococcal infections.

## Introduction

*Staphylococcus aureus*, the Gram-positive opportunistic human pathogen, is the most common causative agent of nosocomial infections, including skin and soft tissue infections (SSTIs), bloodstream infections (BSI), toxin-mediated syndromes, pneumonia, and life-threatening endocarditis as well as community-acquired infections (1). The increasing rate of drug resistance in virulent strains of *S. aureus*, especially methicillin-resistant *S. aureus* (MRSA), is a serious concern in treating and controlling Staphylococcal infections (2). Now, *S. aureus* has become resistant to the broad spectrum of antibiotics, including aminoglycoside, chloramphenicol, tetracycline, β-lactams, fluoroquinolones, and macrolides (3). Although aminoglycosides have potential adverse effects such as nephrotoxicity and ototoxicity, they are still frequently used in combination with β-lactams and glycopeptides to treat Staphylococcal endocarditis and other infections (4,5). Aminoglycosides reversibly bind to the bacterial 30s ribosomal subunit, resulting in the accumulation of truncated or non-functional proteins by derailing translation and misreading the genetic codes (6). 

Generally, there are four major strategies in antibiotic resistance as follows: (i) reducing membrane permeability to the antibiotics either by decreasing uptake or increasing efflux, (ii) drug inactivation due to the hydrolysis or modification, (iii) alteration in drug target and diminished binding permeability, and (iv) mutation (7). Inactivation of the antibiotic by aminoglycoside-modifying enzymes (AMEs) is the main mechanism of resistance to aminoglycoside (8,9). AMEs are encoded by several genes transferring horizontally between the bacterial species resulting in other mechanisms of bacterial resistance (10). Resistance to aminoglycosides is predominantly mediated by five classes of AMEs as follows: aminoglycoside-6'-N-acetyltransferase/2''-O-phosphoryl transferase [AAC(6')/APH(2'')] encoded by *aac(6')*/*aph(2'')* gene; aminoglycoside-3'-O-phosphoryltransferase III [APH(3')-III] encoded by *aph(3')*-*IIIa* gene; aminoglycoside-4'-O-phosphoryltransferase I [ANT(4')-I] encoded by *ant(4')*-*Ia* gene; aminoglycoside-9-O nucleotidyl transferase I [ANT(9)-I] encoded by *ant(9)*-*I* gene, and aminoglycoside-6-O-nucleotidyltransferase I [ANT(6)-I] encoded by *ant(6)*-*I* gene. In Staphylococci, ANT(4')-I, AAC(6')/APH(2''), and APH(3')-III are the most common AMEs affecting tobramycin, gentamicin, and kanamycin, respectively (11). The bifunctional AME *aac(6')*/*aph(2")* confers resistance to nearly all aminoglycosides except streptomycin (12). The *aac(6')-Ie/aph(2")-Ia* (also named *aacA*-*aphD*) gene has been located on the plasmids, transposons such as Tn*4001 *(in *S. aureus*), Tn*5281* (in enterococci), and Tn*4031* (in *S. epidermidis*) and the other mobile genetic elements, increasing the aminoglycoside resistance and the co-resistance to other compounds (13). An increase in high-level gentamicin resistance (HLGR) has been reported in European, Asian, and South American countries. The present study attempted to determine the frequency of antibiotic resistance in clinical isolates of *S. aureus* and genes encoding AMEs and FemA, a cytoplasmic protein essential for the expression of methicillin resistance in *S. aureus* and also involved in the biosynthesis of the staphylococcal cell wall, in aminoglycoside resistant strains in northeastern Iran.

## Materials and methods

### Clinical Isolates and Identification

In this cross-sectional study, from December 2020 to January 2022, a total of 105 *S. aureus* isolates were collected from the different clinical samples such as blood, sputum, urine, wounds, abscess, bronchial washings, and endotracheal secretions from the hospitalized patients in four medical and educational centers affiliated with Golestan University of Medical Sciences, including Sayyad Shirazi, Taleghani, 5-Azar, and Deziani Specialized and Advanced Clinic.

All isolates were cultured on blood agar and mannitol salt agar (Conda, Pronadisa, Spain) and characterized by Gram staining and conventional biochemical tests, such as catalase, coagulase, and DNase activities.

### Antimicrobial Agents and Susceptibility Testing

Antimicrobial susceptibility testing was done using the Kirby-Bauer disk diffusion method on Muller Hinton agar according to the Clinical Laboratory Standards Institute (CLIS, 2020) guidelines. The following antibiotics (Mast discs, Mast Group Ltd, UK) were used: gentamicin (GM, 10 µg), amikacin (AN, 30 µg), kanamycin (K, 30 µg), tobramycin (TM, 10 µg), netilmicin (NET, 30 µg), doxycycline (D, 30 µg), ciprofloxacin (CIP, 5 µg), rifampicin (RA, 30 µg), mupirocin (MUP, 5 µg), teicoplanin (TEC, 30 µg). *S. aureus* ATCC 29213 was used as the quality control strain for in vitro susceptibility testing.

### DNA Extraction and Molecular Detection of the Genes Encoding AMEs

Genomic DNA was extracted from the bacterial colonies by using a commercially available DNA extraction kit (AccuPrep^®^ Genomic DNA Extraction Kit, Bioneer Co. Daejeon, Korea) according to the manufacturer's instructions. The DNA concentration was measured by Eppendorf Biophotometer (Eppendorf, Hamburg, Germany) at 260/280 nm.

The aminoglycoside-resistant *S. aureus* isolates were screened for the presence of the following AMEs-encoding genes: *aac(6')/aph(2''), ant(4')-Ia, aph(3')-IIIa, *as well as for *femA* gene by using specific primers according to the previously described studies (Table 1). The primer sequences were also checked by BLAST service available from the National Center for Biotechnology Information (NCBI) GenBank website (https://blast.ncbi.nlm.nih.gov/Blast.cgi). 

The PCR mixtures were prepared in a final volume of 25 µL containing 12.5 µL of *Taq* DNA polymerase 2X master mix (Amplicon, Copenhagen, Denmark), 10 pmol of each primer, 1.5 µL of DNA template (∼10 ng). Parallelly, the multiplex PCR was also set in a 50 µL reaction mixture comprising 25 µL of *Taq* DNA polymerase 2X master mix, 1 µL of each forward primer, 1 µL of each reverse primer, 1 µL of each DNA, and nuclease-free water to 16 µL. The reactions were carried out in a thermocycler (Bio Intellectica, Canada), and the temperature cycling conditions were as follows: initial denaturation at 94°C for 5 min; 35 cycles of denaturation at 94°C for 1 min, specific annealing temperature (See Table 1) for 45 s, and elongation at 72°C for 1 min; and the final cycle was followed by extension at 72°C for 7 min. *S. aureus* ATCC 43300 was used as the standard strain. The PCR products were electrophoresed in a 0.5% agarose gel containing 0.5 mg/mL ethidium bromide in TBE running buffer at 80 V for 60 min, then were photographed under UV light by a gel documentation system (UVItec, UK). The GeneRuler™ 100 bp Plus DNA Ladder (Fermentas, Vilnius, Lithuania) was used as a molecular size marker.

### Data Analysis

The data was analyzed using SPSS V22.0 (SPSS Inc., Chicago, Ill., USA), and the chi-square test was applied to compare the groups. The P-value of less than 0.05 was accepted as the statistically significant value.

## Results

A total of 105 non-duplicate *S. aureus* clinical isolates were collected from blood (n=12, 11.42%), sputum (n=5, 4.7%), urine (n=2, 1.9%), wounds (n=52, 49.52%), abscess (n=31, 29.52%), bronchial washings (n=1, 0.9%), and endotracheal secretions (n=2, 1.9%). The mean age of patients was 38 years, ranging from 7 to 69 years old, 55.23% (n=58) of patients were male, and 44.76% (n=47) were female. The microbiological analysis of isolates showed golden-yellow colonies on nutrient agar and Gram-positive cocci in grape-like clusters under the microscope. All isolates were biochemically positive for the mannitol fermentation by revealing small colonies surrounded by yellow zones on mannitol salt agar and catalase, coagulase, β-hemolysis, and DNase activities. Overall, 23 isolates out of 105 (21.9%) were found to have MRSA by the disk diffusion method using a cefoxitin disk, and all the MRSA were also positive for the presence of the *femA* gene. MRSA strains were also detected in 9 isolates of aminoglycoside-resistant *S. aureus*. The distribution of MRSA clones in clinical specimens was as follows: wound (n=14, 60.8%), blood (n=5, 21.7%), and abscess (n=4, 17.3%).

### Antimicrobial Susceptibility Testing

**Fig. 1:**
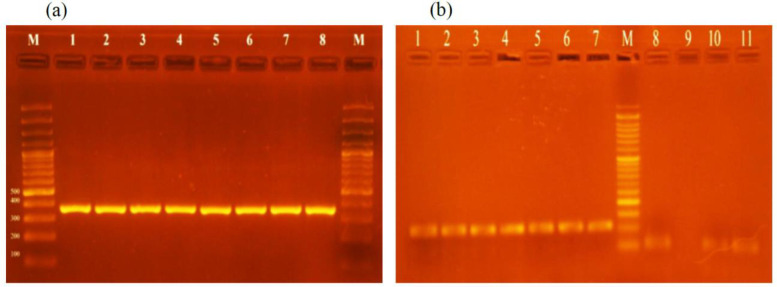
The antibiotic resistance profiles of *S. aureus* isolates

The antimicrobial susceptibility testing revealed that the highest resistance rate was observed for doxycycline (50.4%) followed by ciprofloxacin (49.5%), kanamycin (47.6%), tobramycin (46.6%), amikacin (45.7%), gentamicin (45.7%), rifampicin (35.2%), netilmicin (26.6%), mupirocin (8.5%), and teicoplanin (4.7%) (Figure 1). 16.1% (n=17) and 1.9% (n=2) of isolates were multidrug-resistant (MDR) and extensively drug-resistant (XDR) phenotypes, respectively.

### Distribution of AME Resistance Genes

**Fig. 2:**
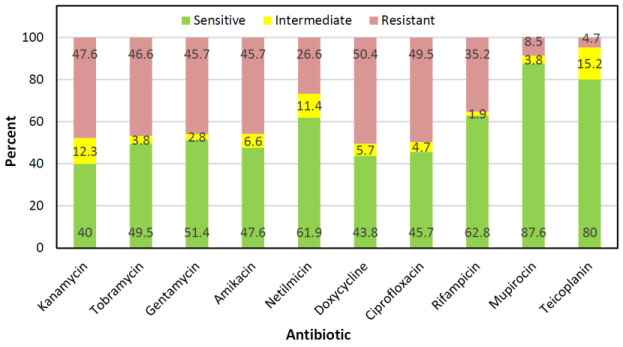
Amplification of *femA* gene in *S. aureus *isolates. Lane M: 100 bp Plus DNA Ladder; Lanes 1-4: *femA* amplicons of *S. aureus *isolates; Lane Neg.C: Negative control.

**Fig. 3:**
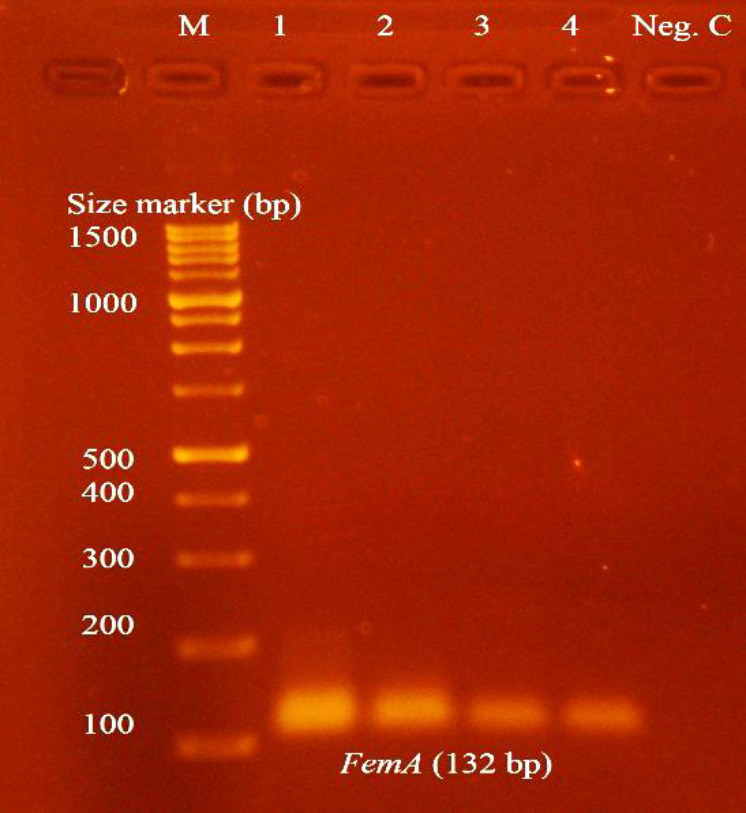
(a). Amplification of *aac(6')/aph(2'') *gene in aminoglycoside-resistant *S. aureus *isolates. Lanes M: 100 bp Plus DNA Ladders; Lanes 1-8: *aac(6')/aph(2'')* amplicons of *S. aureus *isolates (369 bp). (b). Amplification of *aph(3')-IIIa *and* ant(4')-Ia* genes. Lanes 1-7: *aph(3')-IIIa *amplicon of *S. aureus *isolates (269 bp); Lane M: 100 bp Plus DNA Ladder; Lanes 8-11: *ant(4')-Ia* amplicon of *S. aureus *isolates (135 bp).

**Fig. 4:**
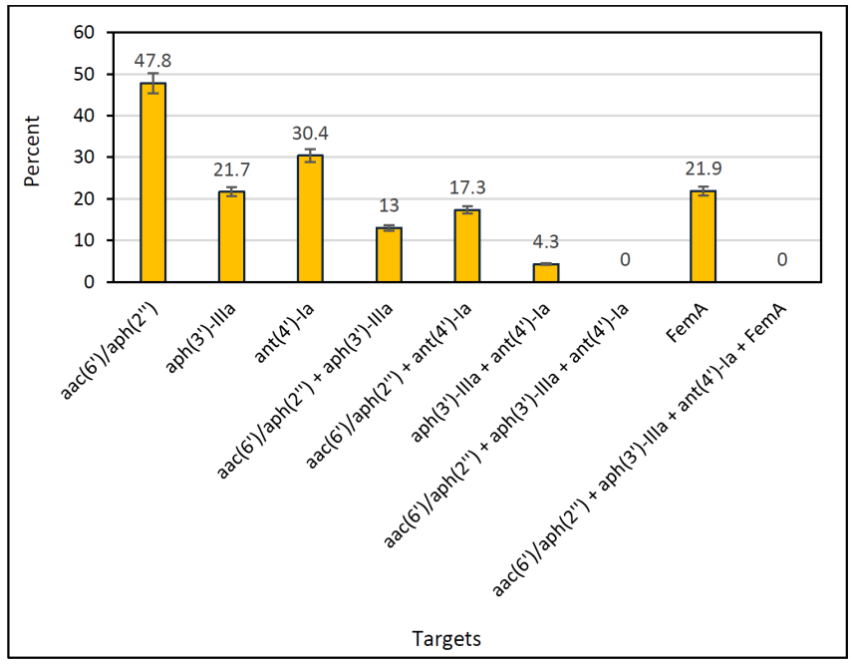
The distribution of the genes encoding AMEs and* femA* gene in *S. aureus* clinical isolates

The clinically relevant groups of aminoglycoside-resistant *S. aureus* isolates were screened for the presence of *femA* and AME resistance genes (Figures 2, 3a, and 3b). 23 out of 105 isolates (21.9%) showed resistance to all the five used aminoglycosides, including gentamicin, tobramycin, netilmicin, kanamycin, and amikacin. The frequency of AME resistance genes was as follows: 11 out of 23 isolates (47.8%) carried *aac(6')/aph(2'')*, whilst *aph(3')-IIIa *and* ant(4')-Ia *were positive in 5 out of 23 (21.7%) and 7 out of 23 (30.4%) of isolates, respectively. The coexistence of *aac (6')/aph (2'') *and *aph (3')-IIIa *was detected in 13% (3/23) of isolates. The AME-encoding genes *aac (6')/aph (2'') *and* ant (4')-Ia *were coexisted in 17.3 (4/23) of isolates. Only 1 isolate (4.3%) harbored *aph (3')-IIIa *and* ant (4')-Ia. *However, a three-plex of *aac (6')/aph (2'')* and *aph (3')-IIIa *and *ant (4')-Ia *was not detected in any *S. aureus* isolates. A significant correlation (*P*<0.05) was found between the aminoglycoside-resistant phenotypes and the presence of genes encoding AMEs. Furthermore, a statistically significant correlation was not observed between the MRSA strains and aminoglycoside resistance in clinical isolates of *S. aureus* (*P*>0.05). The distribution of the genes encoding AMEs and* femA* in *S. aureus* clinical isolates is shown in Figure 4.

## Discussion

In this cross-sectional study, we reported the frequency of genes encoding AMEs and FemA in aminoglycoside-resistant *S. aureus*. Our data revealed the widespread occurrence of AMEs in clinical isolates of *S. aureus* in our therapeutic centers from which the isolates were recovered. Aminoglycoside resistance is mediated through different mechanisms, including enzymatic modification, target site modification by an enzyme or chromosomal mutation, and efflux system. The various members of the aminoglycoside class are affected by the different mechanisms (18). Enzymatic alteration (often via *N*-acetylation or *O*-phosphorylation) of amino or hydroxyl groups of aminoglycosides is the major mechanism of resistance resulting in decreased binding affinity to the ribosome of the aminoglycoside molecule (19). AMEs have been categorized into three groups according to their ability to acetylate, phosphorylate, and adenylate amino or hydroxyl groups found at different situations around the aminoglycoside core scaffold. These three families of AMEs include aminoglycoside *N*-acetyltransferases (AACs), aminoglycoside *O-nucleotidyltransferases* (ANTs), and *O-phosphotransferases* (APHs). The families are also divided into subtypes according to the situation on the aminoglycoside that the enzyme modifies followed by a Roman numeral and sometimes a letter when different enzymes exist that modify the same situation. We found that the *aac(6')/aph(2'')* was the most prevalent AME-encoding gene conferring resistance to aminoglycosides in clinical isolates of *S. aureus*. The high frequency of *aac(6')/aph(2'')*, the most common AME in *S. aureus*, has also been reported in many recent studies (20–24). The resistance to gentamicin, kanamycin, tobramycin, neomycin, and amikacin is often directed by *aac(6')/aph(2'')* although, *aph(3')-IIIa* and *ant(4')-Ia* may also play a significant role in resistance to some types of aminoglycosides (25). The other mechanisms of aminoglycoside resistance such as 16S rRNA methylation and efflux-mediated resistance have also been described in previous studies (10,25). Unlike the AMEs, 16S rRNA methylases have been reported as an emerging mechanism for high-level resistance to almost all clinically important aminoglycosides (26). 

It has been shown that the acquisition of AMEs-mediated resistance determinants in MRSA clones raises the risk of multiple drug resistance and compromises the therapeutic effectiveness of the aminoglycosides group in the fight against *S. aureus* (24,27). Although the standard methods, including cefoxitin disk diffusion, oxacillin-salt agar screening, and molecular detection of the presence of *mecA* gene are currently used for identifying MRSA strains, *femA* has recently been raised as an alternative factor due to the potent conservation in the genome, its key role in cell wall metabolism, and methicillin resistance (28). The *femA*, a chromosomally encoded factor, plays a major role in the last phases of peptidoglycan pentaglycine interpeptide formation by adding the next two glycine residues to the amino group of the lysine side chain in *S. aureus* cell wall. The factor, known as fem (factors essential for methicillin resistance) has shown to be involved directly in methicillin resistance, as well as resistance towards β-lactam antibiotics, and may serve as a target for the development of new antibacterial agents (29). There are many reports on the prevalence rate of MRSA strains isolated from diverse patient populations across the world (30–33). Remarkably, high variability in the prevalence of MRSA has also been reported in multiple geographical regions and even among different hospital wards of similar size and patient case mix. These differences may reflect a combination of various management approaches, infection control policies, surveillance practices, and experiences, the concept of the problem, legislative requirements, and financial and personnel resources. 

In this study, we did not observe any statistically significant difference between the MRSA strains and aminoglycoside resistance in *S. aureus* isolates which is in contrast with some other studies supporting the significant correlation between them (34,35). Moreover, the findings of a study in three tertiary-care hospitals in Greece showed that the rate of erythromycin resistance was statistically significantly lower in methicillin-susceptible *S. aureus* (MSSA) than in MRSA strains (36).

The mobile genetic elements (e.g., insertion sequences, transposons, and sometimes plasmids) carrying antibiotic-resistance genes have also been acquired by MRSA on multiple independent occasions. Resistance to penicillin (*blaZ*), trimethoprim (*dfrA* and *dfrK*), erythromycin (*ermC*), clindamycin (*ermC*), and tetracyclines (*tetK* and *tetL*; *tetM* and *tetO*) have all been recognized on the mobile genetic elements in MRSA strains (32). In addition, AMEs-encoding genes are frequently detected on plasmids containing multiple resistance elements such as other AMEs or β-lactamases. The mobility of the AMEs might be tied to their origins, which has been seen to be through horizontal gene transfer from the actinomycetes responsible for the natural production of aminoglycosides (19). Nevertheless, there is little research on the incidence of AME-mediated aminoglycoside resistance in MRSA and their association with multiple resistance to other antibiotics in the *S. aureus* population in many countries. Further experiments are needed to better understand the function of AMEs, gene expression profile analysis by Real-time PCR as well as the mechanisms driving the dissemination of these genes in MRSA strains.

Several limitations should be taken into consideration when interpreting the findings of this study. Firstly, the sample size proved to be insufficient for accurate statistical measurements. Additionally, our investigation did not encompass the full spectrum of resistance mechanisms related to aminoglycosides. Furthermore, we did not explore the potential involvement of other AMEs-encoding genes in aminoglycoside resistance. Finally, we did not assess the mRNA expression levels of these genes.

**Table 1:** Primer sequences and PCR conditions for amplification of AMEs and femA genes.

Target	Primer sequences (5´→3´)	Annealing temperature	Amplicon size (bp)	References
*aac(6')/aph(2'')*	F: CAGGAATTTATCGAAAATGGTAGAAAAG R: CACAATCGACTAAAGAGTACCAATC	59°C	369	(14)
*ant(4')-Ia*	F: AATCGGTAGAAGCCCAA R: GCACCTGCCATTGCTA	58°C	135	(15)
*aph(3')-IIIa*	F: CTGATCGAAAAATACCGCTGC R: TCATACTCTTCCGAGCAAAGG	55°C	269	(16)
*femA*	F: AAAAAAGCACATAACAAGCG R: GATAAAGAAGAAACCAGCAG	58°C	132	(17)

## Conclusion

Our data reveals that the coexistence of several AMEs and the spread of the resistance determinants like *femA* in MRSA strains are worrying and may contribute to the broadening of aminoglycoside resistance spectra in MRSA. However, it is suggested that the combination therapy with aminoglycosides and other related antibiotics must be intently chosen in staphylococcal infections. This issue requires continuous monitoring of the antimicrobial susceptibility surveillance system and antibiotic stewardship programs (ASPs) to help clinicians minimize losses by optimizing antibiotic prescribing and improving clinical effectiveness.
